# Recent Advances in Electrochemical and Optical Sensing of Dopamine

**DOI:** 10.3390/s20041039

**Published:** 2020-02-14

**Authors:** Faten Bashar Kamal Eddin, Yap Wing Fen

**Affiliations:** 1 Department of Physics, Faculty of Science, Universiti Putra Malaysia, Serdang 43400 UPM, Selangor, Malaysia; faten.mphy@gmail.com; 2 Functional Devices Laboratory, Institute of Advanced Technology, Universiti Putra Malaysia, Serdang 43400 UPM, Selangor, Malaysia

**Keywords:** nanomaterials, electrochemical, optical, surface plasmon resonance, sensors, dopamine

## Abstract

Nowadays, several neurological disorders and neurocrine tumours are associated with dopamine (DA) concentrations in various biological fluids. Highly accurate and ultrasensitive detection of DA levels in different biological samples in real-time can change and improve the quality of a patient’s life in addition to reducing the treatment cost. Therefore, the design and development of diagnostic tool for in vivo and in vitro monitoring of DA is of considerable clinical and pharmacological importance. In recent decades, a large number of techniques have been established for DA detection, including chromatography coupled to mass spectrometry, spectroscopic approaches, and electrochemical (EC) methods. These methods are effective, but most of them still have some drawbacks such as consuming time, effort, and money. Added to that, sometimes they need complex procedures to obtain good sensitivity and suffer from low selectivity due to interference from other biological species such as uric acid (UA) and ascorbic acid (AA). Advanced materials can offer remarkable opportunities to overcome drawbacks in conventional DA sensors. This review aims to explain challenges related to DA detection using different techniques, and to summarize and highlight recent advancements in materials used and approaches applied for several sensor surface modification for the monitoring of DA. Also, it focuses on the analytical features of the EC and optical-based sensing techniques available.

## 1. Introduction

**Figure 1:**
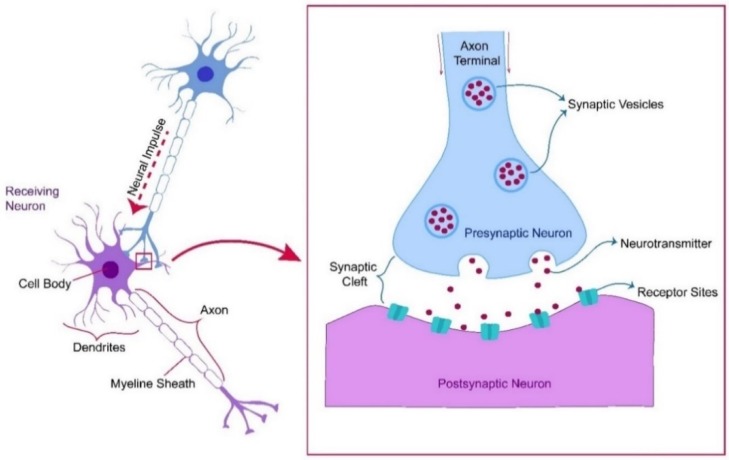
Synaptic transmission process.

In the mammalian brain, neurons carry information throughout the brain to the whole-body using a series of electrical impulses. Signal transfer between nerve cells occurs in two stages, electrically and chemically. Electrical signals are passed along the cell membrane, and then converted into chemical signals carried by small molecules called neurotransmitters (NTs). So, NTs are endogenous chemical messengers secreted by neurons to transmit signals to a target neuron synapse. In a synaptic transmission process as shown in Figure 1, NTs are stored within synaptic vesicles, their role in relaying, adjusting and amplifying signals makes them communicate with muscle cells, gland cells, organs and other neurons. NTs can be categorized according to their functions into an excitatory class which functions to activate receptors on the postsynaptic membrane, and an inhibitory class that functions in a reverse mechanism. The most common types of NTs are acetylcholine, norepinephrine, epinephrine (EP), dopamine (DA), gamma-aminobutyric acid (GABA), glutamate, serotonin (SE), and histamine. NTs were also classified according to their molecular types. DA is classified as monoamine. DA is an important and special NT that possesses both properties, and plays many critical roles in human health related to the central nervous, renal, cardiovascular, and hormonal systems, and other animals including both vertebrates and invertebrates [1,2,3,4,5,6].

In the late 1950s, DA was discovered to be a NT by Arvid Carlsson [7,8]. The adrenal gland and specific areas of the brain are responsible for the secretion and release of DA. DA is the most abundant catecholamine, and as a major neuromodulator, it affects neuronal plasticity, as well as many brain functions. Its role is not limited to that, it controls stress responses, consciousness, information flow and attention span, learning, sleep–wake cycle, motivation, motions, and memory formation. That is to say DA controls brain–body integration [9,10,11,12]. Extreme abnormalities of DA levels in the body can lead to many physiological disorders. An indicator of high DA levels is cardiotoxicity, which in turn leads to rapid heart rate, hypertension as well as heart failure [13]. By contrast, a low concentration of DA may result in some mental and physical diseases such as Parkinson’s disease (PD) [14,15], schizophrenia [16,17,18,19,20,21,22,23,24], Alzheimer’s disease [25,26] and depression [27]. Therefore, the development of highly sensitive and selective sensors to detect DA trace amount in vivo and in vitro is very important in clinical diagnosis, monitoring treatment efficacy, and disease prevention [28]. Concentration levels of NTs can be measured in several biological fluids, such as serum, saliva, urine, plasma, cerebral spinal fluid, and platelets [29,30]. According to the Human Metabolome Database, the physiological levels of DA vary in different human biofluids. The concentration of DA in blood is less than 130 pM, while in human cerebrospinal fluid and urine the levels of DA are 5 nM [31]. Chekhonin et al. concluded that in experimental parkinsonism, the measurement of catecholamines and their metabolites in urine can be considered as a biomarker to evaluate the situation of the dopaminergic nigrostriatal system of the brain [32].

In the performance evaluation of sensors, there are many crucial parameters that should be considered. The most important are the limit of detection (LOD) and the sensitivity which must be sufficient for the concentration level of the target. In addition, the selectivity that must be high enough in the presence of different interfering species in the real sample. Sensor’s precision also includes reproducibility, and this feature proves that the used sensor allows a reproducible measurement in spite of challenges. The sensor repeatability is often directly related to accuracy, where the sensor has the ability to repeat a measurement when put back in the same conditions. Sometimes even an inaccurate sensor can be repeatable during measurements. The probability that the sensor meets the specification requirements is also required, and this is known as reliability. Suitable sensors for clinical diagnostics of DA require detection limits in the order of the nanomolar level.

So far, numerous analytical methods including high performance liquid chromatography (HPLC) [33,34,35], capillary electrophoresis [36,37,38,39], mass spectroscopy [40,41,42], ultraviolet–visible spectrophotometry [43], fluorescence spectrometry [44,45], chemiluminescence(CL) microdialysis techniques [46], Fourier transform infrared (FTIR) spectroscopy [47], flow injection [48], enzymatic methods [49], electrochemical (EC) methods [50,51,52], and other methods have been developed for DA level monitoring. Each method has its own features and drawbacks. Although its selectivity and sensitivity are good with low LODs, most of them have some drawbacks such as consuming time, effort, and money. Moreover, the synthesis of colorimetric and fluorescent probes for DA detection needs complex procedures and steps [53]. To enhance DA sensing performance, several sensor surface modification approaches were undertaken. Also, novel sensors utilizing nanoparticles [54], field-effect transistor (FET) [55], and conducting polymers [55,56] have been developed, but they are still unstable in the long term. Additionally, the surface functionalization of the sensor is complicated. However, EC sensing methods have received increasing attention due to their notable advantages such as high simplicity, sensitivity, good repeatability and reproducibility, long-term stability, and cost effectiveness [57,58,59]. Despite all these features, they suffer some limitations that motivate researchers to make further improvements to enhance their sensitivity, selectivity, and biocompatibility, and reconsider optical based methods such as fluorescence, luminescence, CL, electrochemiluminescence (ECL) and spectrophotometry. These limitations will be mentioned in the following sections. The excellent electrical conductivity, biocompatibility, large surface area, non-toxic properties and low cost of carbon-based nanomaterials have made them receive a great deal of attention. Also, the charge transport properties, EC redox efficiency, high conductivity, facile functionalization and environmental stability of polymers have gained a tremendous amount of interest. This review article presents recent research efforts that have reported significant EC sensing performance towards DA with a focus on using carbon-based nanomaterials and polymers in the construction, modification, and development of the EC sensors surfaces due to their fascinating properties. Many types of optical sensors as alternatives will also be reviewed.

## 2. Electrochemical (EC) Sensors

In the past few decades, many studies using EC techniques have focused on the development of a tool for diagnosis of many brain diseases through the clinical detection of NTs and biomolecules associated with the nervous system. EC techniques in combination with nanotechnology have been excellent models for both in vivo and in vitro quantitative analysis of DA. EC sensors are suitable for the detection of multiple biomolecules. They have attracted a lot of attention due to their favorable features such as simplicity, fast response time, wide linear concentration range, cost effectiveness, real-time detection, possibility of miniaturization, and excellent sensitivity [2,12,52,60,61,62,63,64,65,66,67,68,69,70,71,72,73].

Dynamic EC techniques such as fast-scan cyclic voltammetry (FSCV) [74], differential pulse voltammetry (DPV) [75], square wave voltammetry (SWV) [76], amperometry (AMP) [77], and so forth offer great scope for the determination of DA. In these techniques when an electrode potential is applied, the changes in currents are measured. At specific electrical potentials, DA is easily oxidized on the electrode to DA-quinone, and after that it can be reduced back to DA so it can be effectively measured [78].

To overcome challenges associated with measuring DA in vivo, it is important to understand the environment in which dopaminergic neurons function. These challenges are due to several reasons including that the concentrations involved are usually low, the response time of DA is rapid. Add to that, fast release and remove of DA from the extracellular space [79]. Moreover, what makes the measurement more difficult is the coexistence of several interfering compounds within the biological samples. The most important interfering compounds are AA, UA, and EP. Because at similar potentials, they can all be oxidized and their signals are overlapping so they cannot be separated [80,81,82]. Also, high concentration of AA (~10^3^ times higher than DA), and fouling of the electrode surface increase the difficulty of EC oxidation of DA at conventional electrodes [83]. Consequently, the electrode surfaces will suffer from loss of selectivity, reusability, and reproducibility. Therefore, many attempts have been made to improve the EC DA sensor and overcome limited selectivity, large noise and background signal, and the fouling and degradation of the sensor with time. The focus of the following section is on the development of EC sensors using carbon-based nanomaterials (i.e., carbon nanotubes (CNTs), carbon fiber (CF), graphene and graphene oxide (GO)) and their composites with other materials to achieve simultaneous quantification of DA in real time with stability for long term.

### 2.1. Carbon-Based EC Sensor

Carbon-based nanomaterials in all their forms have been widely used in real-time detection of biomolecules. This is due to their great features such as low cost, biocompatibility, non-toxicity, large surface area, and high electrical conductivity. The properties of these nanomaterials differ depending on their size, diameter, and number of sheets [84,85]. To detect low concentration of DA selectively in the presence of other biological species, the surface modification of carbon-based electrodes including carbon-fiber microelectrodes (CFMEs), carbon paste electrode (CPE), glassy carbon electrode (GCE), glassy carbon paste electrode (GCPE), graphite electrode (GE), graphite paste electrode (GPE), and screen-printed electrode (SPE) appeared as an effective technique to overcome challenges related to the detection electrochemically. Over the past few years, different types of carbon materials, such as graphene, reduced graphene oxide (rGO), single-walled carbon nanotubes (SWCNTs), and multi-walled carbon nanotubes (MWCNTs) have been used. In 2003, modification of GCE with MWCNTs to detect DA and SE using slow-scan CV and DPV was reported [86,87]. The used electrodes are large and the EC measurements cannot cover fast changes. This in turn makes these methods are unsuitable for in vivo monitoring. Unmodified exfoliated GE was used in 2004 by Ramesh et al. They succeeded to observe DA in the presence of 100 μM AA with LOD of 50 nM [88]. The MWCNT-Nafion modified GCE proposed by Kangbing Wu and Shengshui Hu (2004) reduced the potential overlapping and improved the peak current during monitoring of DA [89]. A carbon–polyvinylchloride (C–PVC) composite electrode used by Aguilar and co-workers was stable with good selectivity towards DA available in acidic and in neutral medium with a mixture of AA and UA. The LOD of DA was 0.2 μM [90]. The small size and ease of fabrication of CFMEs as well as good electron transfer features made it widely used. There are many works related to carbon-based EC biosensors which have been undertaken by Venton’s group. For the first time, Swamy and Venton (2007) in their study to co-detect DA and SE in vivo used SWCNTs to modify CFMEs. This treatment aimed to enhance the sensitivity, reduce the biofouling caused by SE and improve electron transfer. Their results demonstrated the potential of this sensor for NTs in vivo monitoring [12]. Chen et al. (2009) modified GCE using MWCNTs, quercetin (Q) and Nafion^®^ to detect DA in human serum samples with the presence of AA. Compared with a bare GC electrode, the results showed improvements in the current response of DA (5-fold) and this increase is due to MWCNTs. Using Q led to a decrease in the oxidation overpotential of DA. Also, Nafion^®^ layer played an active role in promoting selectivity as it prevented the interference of AA with DA. The sensitivity of the batch system was 95.36 mAmol^−1^ Lcm^−2^ with LOD 4.72 µM; while in the flow injection system, the sensitivity reached 121.6 mAmol^−1^ Lcm^−2^ and LOD was 1.4 µM [91].

In the same year, the wonderful material graphene was used for the first time to modify the working electrode for DA sensing due to its extraordinary properties and showed better response than these electrodes modified with MWCNTs [92]. This was followed by numerous studies on the formation of compounds of graphene with metals, metal oxides, polymers, clay, metal–organic frameworks (MOF), carbonaceous, zeolite and other materials which are summarized in Table 1. Xinying et al. (2012) fabricated sensitive and stable graphene-based electrode for DA detection in the presence of AA, EP, and UA in biological and pharmaceutical samples. The sensitivity and selectivity for the modified sensor were excellent with LOD of 5.00 × 10^−7^ M [93]. To detect DA in vivo, CNTs have also been developed to obtain macrostructure CNT electrodes which are called CNT yarns and contain many parallel CNT filaments. The features of these CNT yarns depend in the first place on the nanotubes used to make them, in addition to the twist angle used during spinning. In comparison with conventional CFMEs, CNT yarn disk-shaped (CNTy-D) electrodes reported by Schmidt et al. (2013) to detect DA in rat brain when coupled with FSCV showed improved sensitivity, selectivity, electron transfer kinetics and spatial resolution [64]. Although disk carbon nanotube yarn microelectrodes (CNTYMEs) showed good selectivity towards DA with FSCV, their sensitivity still impairs their use to detect DA in vivo because they are designed with a large surface area. In order to enhance the sensor sensitivity, Yang et al. introduced many approaches on CNTYMEs. By employing the laser treatment approach, the oxygen content in laser-etched CNTYMEs increased which provided more adsorption sites for DA [94]. Also, this treatment created more surface roughness and the edge plane carbon was oxidized to a greater degree. All of this has led to high sensitivity to DA. Despite the satisfactory results of laser etching, it is expensive. This approach requires an optical system set-up and a high-cost laser. So, there is still a need to treat and enhance the properties of the CNTYMEs surface using cheap and easy methods. One of the simple methods used was O_2_ plasma etching, where the microwave plasma system was used with oxygen gas flow [95]. This treatment of the electrode surface improved DA currents and the sensitivity by increasing oxygen containing functional groups on the surface. On the other hand, using an anti-static gun treatment increased the roughness of the surface and thus increased sensitivity to DA. Combining the unique electronic properties of rGO with the attractive catalytic features of Fe_3_O_4_ in a new nanohybrid material to modify the GCE led to enhance the performance sensing towards DA and other analytes [96]. Carbon dots (CDs) and chitosan (CS) composite film was prepared by Huang et al. (2013) to modify the GCE. Using this (CDs–CS/GCE) biosensor for DA monitoring showed the linearity of DA oxidation peak current with DA concentration ranging from 0.1 mM to 30.0 mM, and the LOD value reached 11.2 nM [97]. In the same year, Huang and his team developed the previous biosensor by adding gold nanoparticles (Au NPs) to increase the conductivity of the electrode surface. The Au@CDs–CS/GCE had the highest catalytic activity toward DA oxidation in comparison with the bare GCE, CS/GCE, and CDs–CS/GCE electrodes. The results obtained were promising with LOD of 1 nM [98].

The modification of CFE by graphene flowers (GEF) was reported by Du et al. (2014) for simultaneous measurements of DA, AA, and UA in mouse urine and serum samples. The sensing performance of GEF/CFE was excellent with good sensitivity and selectivity [99]. Figueiredo-filho et al. (2014) modified a GCE with nickel oxide nanoparticles (NiONPs) and CNTs within a dihexadecylphosphate film (NiONP–MWCNT–DHP/GCE) to detect DA and EP simultaneously in human cerebrospinal fluid, human serum and lung fluid using SWV and DPV [100]. The proposed electrode showed persuasive results by DPV particularly during the detection of EP, where the sensitivity was 2.3 times higher than the sensitivity obtained by SWV, the LOD was 6.0 times lower than SWV LOD. Add to that for DPV, the linear concentration range verified was lower than that for SWV. During DA detection, the linear concentration range was the same using both techniques, although the SWV sensitivity was 2.1 times higher than the value obtained by DPV and the LODs were very close. The separation of the reduction peak potentials for DA and EP using DPV was good (about 360 mV), and the detection limits obtained were very low: DA (5.0 × 10^−8^ M) and EP (8.2 × 10^−8^ M). The effectiveness of this novel EC method was proved for measuring the concentration of DA and EP in real samples. During the same year, new materials have been hybridized to improve the sensing performance of the GCE by Cincotto et al. (2014). They used the sol–gel process to modify mesoporous silica (SiO_2_) with GO, then decorated it with silver nanoparticles (Ag NPs) [101]. The results showed that electrode modification with Ag NPs/SiO_2_/GO increased the sensitivity of the sensor towards DA and EP, and demonstrated the potential of this sensor to detect DA in real samples with LOD of 0.26 μM without any significant interference from other biological species. Also, Wang el al. modified the GCE using Au nanoplates and rGO. Their results showed that the morphology of Au NPs on the modified electrode had an effect on its selectivity and sensitivity. The Au/rGO/GCE had the largest effective surface area. The excellent electron transfer property of rGO on the electrode surface increased the electrochemical active sites, which led to the enhancement of electrocatalytic reaction toward the oxidation of AA, DA, and UA. The modified electrode showed stability, reproducibility, good anti-interference ability with LOD of 1.4 μM [102]. Yang, Y.J. and Li, W. (2014) used hexadecyl trimethyl ammonium bromide (CTAB) functionalized GO/MWCNTs to modify GCE. The new hybrid material (CTAB-GO/MWCNT) increased the sensor’s surface area, and improved its performance. The linear response range of DA was 5.0–500 μM and the LOD value was 1.5 μM [62]. Nan-Sen et al. developed and designed a wearable, compact, wireless, battery-powered EC device. This fabricated sensing system was used to monitor DA in real time. Where a CFME was surgically fixed in the caudate putamen area of rat brain. DA solution was recorded in the concentration range of 0.5 × 10^−6^–7.0 × 10^−5^ M by employing FSCV method [103]. Another study showed that using Nafion and poly (3,4-ethylenedioxythiophene) (PEDOT) to modify CFMEs is appropriate. The modified electrodes had mechanical stability and durability. Their sensitivity and selectivity toward DA were high [104].

Solid carbon nanopipette electrodes (CNPEs) were fabricated to detect DA in *Drosophila melanogaster*. These sharp electrodes with small diameter tips (approximately 250 nm) were tested firstly in vitro for different concentrations of DA. Then were used to detect endogenous DA release in *Drosophila* larvae, which proved that CNPEs could be suitable and better than CFMEs for NTs measurements in vivo in cases of small organisms (e.g., *Drosophila* brain) [105]. Zestos et al. (2015) have grown carbon nanospikes (CNS) on metal wires such as tantalum (Ta), palladium (Pd), niobium (Nb), and nickel (Ni) using chemical vapor deposition method (CVD) to develop an EC sensor for DA detection [106]. The results of their work proved that carbon played an important role in enhancement of the sensitivity, selectivity, and the LOD of pure metal wire. After exposure all CNS coated metal wires to 1 µM DA solution, their oxidation and reduction peak was clear; while the sensitivity of all metal wires was not enough to detect 1 µM DA using FSCV. Also, different FSCV results were given for DA, UA and AA. A good selectivity of CNS-Ta sensor has been successfully demonstrated. Tang et al. (2015) modified the acupuncture needle (AN) with Au NPs on the tip surface, then they deposited the graphene using the EC method for DA monitoring [107]. The modified (G-AN) significantly increased the sensitivity compared to the bare needle because the presence of Au NPs increased the surface area, and the graphene improved the conductivity of this sensor. Modification of the GCE using multilayer graphene nanobelts (GNBs) as an active layer was reported by Kannan et al. (2016) for the first time [108]. Using GNB/GCE in EC sensing of DA in the presence of the interfering compounds showed a high sensitivity value 0.95 μA μM^−1^ cm^−2^ with LOD value of 0.58 μM. Atta et al. (2016) fabricated ultrasensitive sensor by a mechanical casting method. The cyclodextrin (CD)/ionic liquid (IL) crystal/graphene composite electrode was used to detect multiple NTs including DA, EP, SE, and norepinephrine [63]. The sensor sensitivity was increased because of graphene due to the high electrical conductivity and large surface area. The LOD for EP was as low as 10 pM. To detect DA and UA simultaneously, Zhang et al. (2016) modified GCE with poly (L-lysine) (PLL)/GO. The selectivity of the reported modified electrode was excellent. It showed high sensitivity, long-term stability, and good reproducibility. The LOD value for DA was 21 nM. This novel sensor also showed satisfactory results when used to analyze DA and UA in human blood serum, urine and DA hydrochloride injection [109]. Yang et al. (2017) developed different protocols to fabricate CNT fibers by wet spinning using several materials such as polyethylenimine (PEI/CNT), and chlorosulfonic acid/CNT [110]. Also, they produced CNT yarns which had abundant oxygen content and improved the surface roughness. Each protocol used to prepare these CNT fibers played an important role in altering the fibers surface structure, which in turn improved the EC sensing towards DA and SE.

Aoun (2017) for the first-time used nitrogen-doped graphene quantum dots (N, GQDs)–chitosan nanocomposite (CS) to modify the nanostructured screen-printed carbon electrode (SPCE). The novel CS/N, GQDs @SPCE sensor was employed to detect DA in human urine [111]. Choo et al. (2017) developed a 3D porous graphene oxide (pGO) with Au NP composites for DA measurement [112]. An ultrasonic probe was used to prepare pGO then Au NP was incorporated and 3D pGO-Au NP-pGO-modified indium tin oxide (ITO) electrodes were reported as a novel platform to detect DA electrochemically. Using CV measurement, the reported sensor sensitivity for DA was excellent, and the LOD was 1.28 µM with linear response in the range from 0.1 µM to 30 µM. With regard to selectivity, after adding 10 µM of DA the current change of 8 nA was obtained; while after the same concentrations of glucose and AA were added, no significant change in the signal was observed. Atyah et al. (2017) prepared a novel, simple, low-cost EC sensor to detect DA in the presence of AA and UA with high sensitivity and selectivity using a sonogel-carbon (SNGC) electrode which was modified with L-histidine (L-His SNGC) [113]. The electrocatalytic behavior of this innovative electrode towards DA oxidation was great in human serum and phosphate-buffered saline (PBS). And the simultaneous monitoring results of DA, AA, and UA in pH 7.4 PBS were excellent and the LOD was 1 × 10^−7^ M.

Demuru et al. (2017) developed the surface of CFMEs by using electro-polymerization of Nafion perfluorinated resin and 3,4-ethylenedioxythiophene (EDOT) with two surfactants such as sodium dodecyl sulfate (SDS) or sodium dodecyl benzene sulfonate (SDBS) to synthesize coatings [114]. The surfactants used increased the solution conductivity and decreased the surface tension at the electrolyte electrode interface. The obtained results showed that the PEDOT: Nafion-SDS and the PEDOT: Nafion-SDBS coatings have unique features which made them one of the best coatings ever used for DA determination in the presence of AA, 3,4-dihydroxyphenyl acetic acid (DOPAC), SE and adenosine. There was a marked increase in sensitivity, and the selectivity was good. SDS facilitated Nafion incorporation in the polymeric matrix and as a result, the AA signal is decreased compared to the bare carbon, and enhanced the PEDOT conductivity with the addition of sulfate groups, hence the higher sensitivity. However, SDBS replaced Nafion completely in the polymer matrix and enhanced PEDOT conductivity by adding sulfonate groups, which in turn enhanced absorption of DA on the surface. The improvements done on the CF increased the DA signal by 4X–9X in comparison with bare carbon, and the sensitivity reached 34.4 ± 14 nA/μM (5X) when SDBS was used. In the next year, Demuru et al. (2018) fabricated for the first-time glassy carbon nanorods, and demonstrated that this nanostructured array (with more than 6000 electrodes) enhanced the current density, and the sensitivity of DA with low concentrations in comparison with carbon microfibers. The detection limit was 60 nM [115].

The work presented by Tan et al. (2018) has added a lot to key metrics of DA microsensor including selectivity, sensitivity, detection limit and signal to noise ratio [116]. They demonstrated the role of the hybrid microelectrode in improvement the sensing performance of DA microsensor in the presence of SE and AA. Electrophoretic deposition (EPD) method was used to microfabricate hybrid MWCNT films modified boron-doped ultrananocrystalline diamond (UNCD) microelectrodes. Changing the film microelectrode thickness in this novel EC microsensor led to excellent results were the best at the thickness of 100 nm where the sensitivity was 36 µA/µM/cm^2^ (>125-fold) with a linear range of 33 nM to 1 µM and the LOD of 9.5 nM (>180-fold). Ding (2018) developed a highly sensitive and selective amperometric sensor for DA detection based on electrocatalytic activity of graphene-based macroporous carbon aerogel microelectrode [70]. The synthesis of the three- dimensional carbon aerogel electrode (3D CAG) was done by freeze drying of graphene and MWCNTs together with Nafion. The reduction of 3D CAG improved the electrode conductivity and surface area. The obtained results demonstrated the potential of 3D rCAG sensor in DA investigation in human serum samples. The EC response of this sensor was sensitive and rapid with a LOD of 30 nM. In the same year, Krishna et al. used a copper–molybdenum (Cu–Mo) impregnated α-Al_2_O_3_ NPs catalyst to synthesize fiber-like carbon nanotubes (f-CNTs). The modified electrode (f-CNTs/GCE) showed enhanced sensitivity towards DA with LOD of 5.3 μM [2].

Fayemi et al. (2018), developed stable EC sensor by modify the GCE using polyaniline (PANI)-MO (where MO are NiO, ZnO, and Fe_3_O_4_ NPs) nanocomposites coating to detect DA in the presence of AA and SE [73]. To determine the EC response of DA, DPV was used at physiological pH 7.0. DA determination dynamic range was from 2.0 × 10^−5^ to 2.4 × 10^−6^ M with LOD of 0.153 × 10^−7^, 0.166 × 10^−7^, and 0.176 × 10^−7^ M for GCE/PANI-NiO, GCE/PANI-ZnO, and GCE/PANI-Fe_3_O_4_ sensors, respectively. According to the LOD value, it was clear that the best electrode is GCE/PANI-NiO. The selectivity of these sensors was satisfactory. For the first time, Xu et al. (2018), fabricated a novel, and highly selective EC sensor for DA detection in the presence of other analytes using CV, and DPV techniques based on a (PEDOT) modified laser scribed graphene (LSG) [117]. The LSG electrodes were produced with a 3D macro- porous network and large electrochemically-active surface area by direct laser writing on polyimide sheets. The anodic peak current obtained by the PEDOT-LSG electrode was significantly higher in comparison with bare LSG, and the voltammetric peak separation for DA, UA, and AA was improved. At PEDOT–LSG, the DA detection linear range was from1 to 150 µM with a sensitivity of 0.220 ± 0.011 µA μM^−1^, and a LOD of 0.33 µM; these values are better than others that were obtained by bare LSG. As known to all, the presence of residual oxygen-containing functional groups in rGO makes it highly dispersed. After reduction, the conductive carbon-conjugated networks can be restored, and this leads to increased electrical conductivity in rGO compared to GO. Recently, integrating MnO_2_ NWs with electrochemically reduced graphene oxide (ERGO) have been successfully done by He et al. (2019). They modified GCEs using MnO_2_ NWs/ERGO nanocomposites for ultrasensitive EC detection of DA. Three linear ranges (0.01–0.10 µM, 0.10–1.0 µM, 1.0–80 µM) were obtained on this promising sensing platform, with LOD of 1 nM. The proposed sensor showed high accuracy and good recovery when it was applied to detect DA in human samples [118]. Table 1 summarizes some recent studies that used carbon-based material as a sensing platform for DA quantification.

### 2.2. Polymer-Based EC Sensor

Polymers have unparalleled properties that make them attract attention [194,195,196,197]. Their EC properties qualify them to be used as active sensing materials for biosensors. Add to that, it is easy to synthesis them. The interest that conducting polymers have received has been caused by their excellent electrical conductivity and biological compatibility. They have the ability to increase the redox process rate during measurements. Polymer coatings were commonly used with carbon-based electrodes owing to their important role in resistance the electrode surface fouling. This combination led to composites with enhanced conductivity and improved mechanical strength [198,199]. In NTs detection, non-conductive polymers also have been used to develop sensors due to their insulating properties that reduce the EC signal resulting from other biological interferences. This in turn improves the selectivity of the sensors [200]. However, the electrode modification is necessary because the bare electrode cannot distinguish between DA and other biological samples due to the overlapping of their signals. Many studies have demonstrated the potential of the polymeric films to reject the negatively charged AA.

The overoxidized polypyrrole (OPPy) (dodecy1 sulphate) film-coated GCEs (OPPy (DS) /GCE) succeeded to detect DA with LOD of 40 nM. It showed enhanced selectivity with good reproducibility after multiple measurements [201]. The overoxidized film of a polypyrrole (PPy) derivative was reported by Arrigan (1997). The modified GCE with the coating showed enhanced selectivity towards DA using the CV method [202]. Yuan et al. (2001) demonstrated that using a poly (2-picolinic acid) to modify GCE improved the detection of DA. The modified electrode detected DA down to 30 nM [203]. In the same year, Wu et al. (2001) reported that GCE modified with an over-oxidized poly (N-acetylaniline) (PNAANI) is effective to detect DA selectively with LOD of 16.8 nM [204]. After that, Domenech et al. (2002) showed that the response of EC oxidation signals of DA was enhanced when zeolite-Y-encapsulated 2,4,6-triphenylpyrylium ion (TP^+^) was used to modify the polymer film electrodes (PFEs) which deposited over GCE and graphite/polyester composite electrodes [205]. The deposition of poly-(1,2-phenylenediamine) (OPPD) on CFMEs was undertaken by Mo and Ogorevc to detect DA in the presence of AA [206]. Using CV after overoxidation, the current response of OPPD-CFME was high to cationic DA. The LOD of DA in the presence of AA was 10 nM and in the absence of AA was 2 nM. Rubianes, M., and Rivas, G. (2003) modified different carbon materials including GC, GCP, graphite, graphite paste, CF and SP electrodes using a melanic polymer. The sensing performance of the modified electrodes towards DA in the presence of AA was studied, and their responses depended strongly on the used carbon material [207]. As one attempt to develop voltammetric methods to measure DA with other samples, Raoof et al. (2005) used functionalized PPy films with ferrocyanide (FCN) to modify CPE [208]. The EC behavior of the ferrocyanide modified carbon paste electrodes (PPy/FCNMCPEs) was studied using CV, DPV, and LSV methods. This modification of the electrode surface separated the oxidation anodic peaks of DA and AA very well.

Copper (Cu)-(3-mercaptopropyl) trimethoxy silane (MPS)-complex modified electrode (Cu-MPS) was successfully developed to detect DA [209]. Nafion thin film was coated on the surface of the modified electrode and contributed to neglect the signals related to other biological species. Also, it was responsible for the sensor stability. The electrocatalytic activity of the proposed electrode during DA determination was good and the LOD was 50 nM. The incorporation of fibrous poly (neutral red) (PNR) on functionalized MWCNTs (f-MWCNTs) was done to modify the surfaces of GC, ITO, and gold electrodes [210]. The catalytic activity of f-MWCNTs-PNR composite film on DA and other biological compounds was good. They replaced PNR by poly (methylene blue) (PMB) which led to increase the electron transfer rate, enhancement of the sensitivity and the functional properties [211]. Abdelwahab et al. (2009) developed a sensor to detect DA in vivo by incorporating Cibacron Blue (F3GA) into poly-1,5-diaminonaphthalene (PDAN). This rapid sensor was tested in human urine samples and showed high stability and selectivity [212]. By using Ag/PANI composite nanotubes to modify ITO electrodes, the electrocatalytic activity for oxidation of DA was improved [213]. In another study, Prakash et al. (2009) used PANI with poly (diallyldimethylammonium chloride) (PDDA) and gold (Au(0)) NPs to formulate ternary nanocomposites which were adsorbed on GCE. These composites contributed to the sensing of low concentrations of DA down to 0.05 mM [214]. In addition, several other Au-PANI based electrodes were prepared and employed to monitor DA with more sensitivity [215,216]. The use of two conducting polymers; poly [N-(2-cyanoethyl) pyrrole] and poly (N-methylpyrrole) to prepare ultrathin films for GCE modification was reported by Fabregat et al. (2011). Although the addition of Au NPs has contributed to enhance the sensing performance of the polymers used towards DA, it has not been so important to affect their response to DA [217]. The development of Au NPs-coated PEDOT polymer modified gold electrode in presence of SDS to determine DA selectively was reported by Atta et al. (2012). They also demonstrated the validity of this novel sensor to detect DA electrochemically in human urine samples [218]. Blue-4 (RB4) dye entrapped PDAN was used to modify GCE for DA measurements in the presence of acetaminophen (AP) [219]. Xu et al. (2013) reported the development of PEDOT doped with CNTs (PEDOT/CNTs) on CPE. The proposed sensor showed excellent catalytic property, stability and the complete absence of any interference from AA that was present at higher concentrations than DA [220]. In Sasso et al.’s work (2013), the modification of gold electrodes with overoxidized doped PPy and a poly (sodium 4-styrenesulfonate) (PSS) doped electropolymerized was presented. The sensitivity enhancement of DA released from PC12 cells was demonstrated [221]. Fabregat et al. (2014) in their study used two different conducting polymers, PEDOT and poly (N-methylpyrrole). The amount and morphology of the used polymers with perfectly controlled coating thickness enhanced the sensor response and sensitivity towards the oxidation of DA [222].

A ferrocene-functionalized (PEDOT-Fc: PSS) layer was prepared on an ITO electrode for the amperometric detection of DA. This was done in two stages, the electrodeposition of PEDOT-N3 was followed by copper-catalyzed azide–alkyne cycloaddition of ethynylferrocene. The sensitivity of the proposed sensor was 196 mA M^−1^ cm^−2^ with LOD of 1 µM [223].

In the work reported by Mir et al. (2015), a novel conducting polymer nanocomposite film was used to develop a simple amperometric nano biosensor with high sensitivity and selectivity to determine K^+^ -induced DA released from dopaminergic cells (PC12) [224]. The electro polymerization of Nafion and PEDOT containing composite polymer on CFMEs has been done by Vreeland et al. (2015) in order to enhance the mechanical stability of the electrodes and to increase the sensitivity and selectivity of the proposed sensor. After implantation of the coated electrodes in the brain of male rats for 6 h, they did not lose their selectivity. This improvement is what distinguishes it from uncoated electrodes [104]. Also, electrodeposition of PEDOT/GO onto the surface of CFE led to enhanced sensitivity towards DA with lower LOD. The thickness of the prepared coatings affected the adsorption, sensitivity, and electron transfer kinetics [225]. Graphene and poly 4-amino-3-hydroxy-1-naphthalenesulfonic acid modified SPC sensor was used to monitor DA and 5-HT simultaneously in human urine samples, blood and pharmacological samples [226]. Raju et al. (2019) have combined waveform modification with polymer coating to improve the selectivity of DOPAC and distinguish it from DA. During this study, FSCV was applied and CFMEs were modified and functionalized with PEI and Nafion. The results showed that the CFME modified using electrodeposited Nafion electrostatically attracted DA, but repelled anions such as DOPAC, UA, or AA due to the negative charge of Nafion coatings. Conversely, CFMEs coated with PEI electrostatically attracted the negatively charged anionic DOPAC. This is because the protonation of the nitrogen functionalized groups applied a more positive charge to the electrode surface when PEI was electrodeposited [227].

This is a brief review of some studies that used polymers in EC detection of DA. Further studies are summarized in Table 2.

## 3. Optical Sensors

Besides EC methods, optical detection approaches have appeared as promising sensing techniques especially for in vivo measurements of NTs. Optical sensors have proven themselves strongly because of their features. Their reproducibility and sensitivity are often high. In addition, their detection limit is often in the range of nanomolar or less [1]. Also, using the optical spectrum in a wide range can minimize the interference caused by other biological compounds. Furthermore, because the optical signals are transmitted through the cables of the fiber optic, there is no need for electrodes and electrical wires during the implantation of the probe to get a signal. Since the release and uptake of NTs occur in a fast mechanism, so the focus on temporal and spatial resolutions should be given the same importance during in vivo measurements of NTs [79].

**Figure 2:**
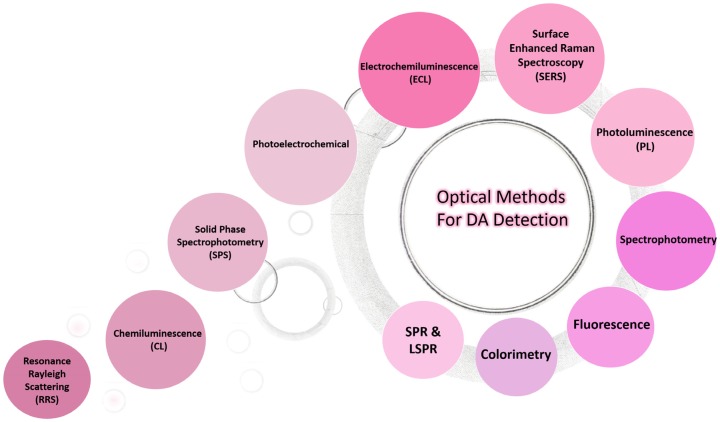
Different optical methods used to detect DA.

In recent years, various optical methods have been used for the detection of DA including colorimetry and spectrophotometry [278], fluorescence [279,280,281,282], ECL [283], surface-enhanced Raman spectroscopy (SERS) [284,285,286,287,288,289], chemiluminescence (CL) [290], photoelectrochemical (PEC), photoluminescence (PL), solid phase spectrophotometry (SPS), resonance Rayleigh scattering (RRS), and surface plasmon resonance (SPR) spectroscopy [291,292,293,294,295]. Figure 2 shows several optical methods were used to detect DA. These methods have their own advantages and disadvantages and often suffer from limited sensitivity and selectivity. However, despite the multiplicity of optical methods used, there is still a great challenge in the biomedical field to develop methods and techniques for highly sensitive and selective detection of DA.

### 3.1. Colorimetry and Spectrophotometry

Colorimetric detection of biomolecules is rapid, simple, and cost-effective. The detection can be performed directly by observing the color change precisely using ultraviolet–visible (UV−vis) spectroscopy, or even with the naked eye. The unparalleled physical and chemical properties of nanomaterials and their abilities to accommodate multiple functional groups and target the biomolecules qualified them to be widely used as excellent components in biological sensing [296,297]. AuNPs are among the most commonly nanomaterials used for the application of biosensor due to their unique optical, physical, and chemical properties. Their surface-to-volume ratio is high effective, their absorption in the visible and near-infrared (VNIR) portion of the electromagnetic spectrum is strong. Also, AuNPs have high chemical stability and excellent electrocatalytic properties. Moreover, what makes these especially plasmonic nanoparticles distinctive is that they are inert under physiological conditions [298,299,300,301].

Owing to the unique optical and chemical properties of AuNPs and AgNPs, they have been widely used as a colorimetric assay platform for DA and various analytes without the use of advanced instrumentation based on the SPR extinction changes of these NPs. The morphological sharpness, diameter, and aggregation status of the AuNPs strongly affect the SPR. The color changes of AuNPs are robustly associated with the reversible process from their dispersion to their aggregation and can be observed by bare eyes and quantified using UV-Vis spectroscopy. As well as, the SPR is highly sensitive to the medium in which AuNPs are dispersed and the interparticle distance. In the context of DA detection, DA has two equivalent hydroxyl groups that form hydrogen bonding between DA and surface modifiers on the AuNPs. This in turn induces the aggregation of AuNPs. This was explained by Chen et al. in their research to develop a probe to detect DA quantitatively in spiked serum at concentrations as low as 33 nM using melamine (MA)-modified AuNPs based on the aggregation of the AuNPs [302]. AuNPs were aggregated and their color changed from red to blue when the free exocyclic amines of MA reacted with them. In the presence of DA, DA was conjugated to MA via hydrogen bonding and inhibited the MA-induced aggregation of AuNPs. This in turn changed the color from blue to red.

The aggregation of several NPs based on interactions other than hydrogen bonding was employed for colorimetric detection of DA [303,304]. As previously mentioned, AgNPs have also been used for the colorimetric sensing of DA. AgNPs show many advantages over AuNPs. These advantages include high extinction coefficients, sharp extinction bands, high scattering-to-extinction ratio, and extremely high near-field enhancements [305]. However, the chemical stability of AgNPs is poor and their investigated surface chemistry is less than those of AuNPs. This in turn makes their applications limited. In addition to AuNPs and AgNPs, other nanomaterials were also used for the colorimetric monitoring of DA. Wang et al. (2013) reported a magnetic Fe_3_O_4_-based biosensor with AuNPs as colorimetric probes for the first time. Using this sensor, the LOD of 10 nM for DA was obtained [306]. The sensitivity of DA sensors that use AgNPs and other NPs is less than that of those that use AuNPs. Despite all the achievements so far, colorimetric sensing systems based on specific molecular interactions of NPs with DA still have drawbacks and need further development to improve the sensitivity and selectivity.

### 3.2. Surface-Enhanced Raman Spectroscopy (SERS)

SERS has gained popularity in biosensing owing to its unique properties such as ultrahigh sensitivity and selectivity, rapid response, high structural specificity, minimal sample preparation, high flexibility and amenability to molecular fingerprinting. This powerful analytical technique is commonly applied to amplify and provide orders of magnitude increases in Raman intensity of Raman active analytes. SERS uses nanoscale roughened metal surfaces and employs the idea that molecules illumination by fixed-frequency light resonantly drives the surface charges creating localized surface plasmons. Typically, VNIR are used to excite Raman modes. Since the plasmon resonance frequencies of Au and Ag fall within these wavelength ranges providing maximal enhancement for VNIR light, they are primarily used with SERS sensors. When the used laser light is matched to the absorption maxima of the molecule, further amplification of the Raman signal occurs. In the sensitivity determination of the sensing system, the conjugation of DA to the SERS substrate is considered an important factor. To avoid sample damage, the wavelength of the laser used and the duration of the illumination should be determined carefully. An et al. (2011) reported that a SERS-based immunosensor is a promising tool to examine the association of antibodies to DA that were both covalently bound and adsorbed to AuNPs deposited on a glass substrate surface. They obtained reproducible wavelength-scanned SERS measurements in a wide spectral range using the same substrate. Raman signal of DA was detected based on antigen-antibody interactions with LOD of 1 ng/mL [307]. The developed sensor by Lim et al. using a CS-Au nanoshell as a SERS substrate was very simple [308]. The results showed that the intensity of the Raman peak near 1382 cm^−1^ increased by increasing DA concentration. The LOD of 1 mM is not satisfactory for practical applications. In another study, they used CS-Au nanocomposites combined with optical fibers as SERS substrates to detect DA levels. Amplified Raman signals of DA were obtained in the dynamic range 1–10 mM [309]. The sensitivity of Raman signals to DA levels was linear. Also, the LOD of this fabricated sensor was not satisfactory.

Many studies demonstrated that using Au and Ag nanostructures to amplify the signal enhances the sensitivity of the sensors. Also, combining different types of materials each having Raman enhancement effect improves the sensitivity and has great potential for practical applications.

### 3.3. Fluorescence Spectrometry

The special optical and photophysical properties of quantum dots (QDs) with respect to their excitation and emission spectra, their quantum yields, biocompatibility and solubility in water made them widely used in biosensors [310,311,312]. QDs are much more photostable than most fluorophores so they have been used in several assays to detect DA sensitively in addition to other fluorescent nanomaterials such as CDs, gold nanoclusters (Au NCs), Au NPs, silica NPs, polymer NPs, and CNTs. In fluorometric DA detection, the interaction with DA in the sensing system leads to change in the fluorescence intensity and this change is used as the basis for the monitoring. In general, the sensitivity of fluorescence-based sensors for DA detection is high. The best detection limit of 0.1 pM was achieved using functionalized CDs with boronic acid and amino groups [313].

### 3.4. Electrochemiluminescence (ECL) Spectrometry

ECL is a kind of luminescence produced by electrode reactions. This powerful technique has been used for the sensitive detection of DA. It combines electrochemistry and CL. In the ECL process, the chemical species generated on the electrode surface undergo high-energy electron transfer reactions to form excited states that emit light after decay to ground state. The immobilization of a luminophore on the electrode surface plays a very important role in ECL detection based on the quenching effect. For DA sensing, a variety of luminophores were used such as QDs, Ru (bpy)_3_^2+^, luminol, peroxydisulfate, noble metal nanoclusters, carbon and other nanostructures. The obtained LODs of ECL sensors were in the pM range when surface-modified QDs [314], g-C_3_N_4_ NSs [315], and TiO_2_ NPs [316,317] were used as luminophores. The fabrication of GDs based sensor is complex compared to other sensors using g-C_3_N_4_ NSs and TiO_2_ NPs. In the case of sensors that employ noble metal nanoclusters, the fabrication is very simple, but the sensitivities need enhancement. Direct determination of DA in blood was done using NIR QDs as ECL materials with very poor selectivity. So, the development of convenient techniques is needed to overcome challenges related to direct quantification of DA in human samples. The sensitivity and selectivity of the SERS-based DA sensors are higher in comparison to other detection techniques. But for analysis, they require expensive equipment and this is an obstacle to the availability of these sensors.

### 3.5. Surface Plasmon Resonance Spectroscopy

As powerful analytical tools that have enjoyed widespread attention over the past two decades, SPR sensors have been developed in several configurations to detect several analytes. These sensors serve in different fields such as medical diagnostics [318,319,320,321,322], food quality [323,324], environmental protection [325,326,327,328,329,330,331] and others. SPR, a refractive index-based detection technique has emerged as a promising sensing platform for sensitive detection of NTs, especially DA due to its substantial advantages. Incorporation a variety of nanomaterials into the sensor chip enhanced the signal and pushed the detection limits to lower values. SPR sensors offer significant advantages such as direct label free detection, high reliability, real-time analysis, very high sensitivity with low detection limit, long-term stability, cost-effectiveness, suitable size, easy sample preparation, the need for a small sample, and reagent consumption. Another feature of SPR sensor is that it is reproducible, this was reported by Kumbhat et al. [332]. In their work, DA and bovine serum albumin (BSA) protein (DA–BSA) conjugate was immobilized onto the surface of gold chip by physical adsorption. Their results showed that the surface regeneration was highly effective and the affinity reaction of DA–BSA conjugate with DA receptors (DA-RC) remained highly reproducible with ≥94% recovery for not less than 25 cycles of measurements. The lowest detection limit achieved in DA sensing using SPR technique was 200 fM [31]. So far, the use of SPR technique to detect NTs in general and DA in particular is still limited. This is encouraging for further research and studies to improve its performance and sensitivity by functionalization the gold surface using advanced materials to detect NTs precisely and overcome all drawbacks.

Table 3 shows, summarizes and compares examples of advanced materials that were used to fabricate and develop DA sensors based on optical methods.

## 4. Conclusions

The clinical sensing of NTs and biomolecules related to the nervous system with high sensitivity and low-cost provides a deeper understanding of the chemical reactions that occur in the brain and offers essential information about human health, which might be employed as a powerful diagnostic tool for different mental disorders. Recently, using nanomaterials in the sensing systems due to their unique properties has been reported in many published works. The numerous advantages offered by EC and optical sensors made them the two most commonly applied methods for both in vitro and in vivo determination of DA. However, the simultaneous detection of DA in the presence of several interfering molecules still remains a fundamental challenge for both techniques. The promising features of carbon-based EC sensors have made their use dominant in the DA sensing field. Polymers have also been widely used to enhance the biocompatibility and the redox properties of the EC sensors. There are many features of EC sensors that made it attracts attentions such as simplicity, fast response time, wide linear concentration range, cost effectiveness, real-time detection with suitability, and excellent sensitivity. It could be deduced that the carbon-based nanomaterials and polymers enhanced the sensitivity of EC sensors towards DA. The EC sensor that was fabricated using graphene to support the synthesizing of Pd NPs by employing supercritical CO_2_ fluid (scCO_2_) provided the highest sensitivity to DA (287 µA/µM). Three-dimensional reduced graphene oxide-based DA sensor has been evidenced to exhibit high sensitivity of (244.17 mA/mM). The simultaneous EC detection of DA on a poly (L-lysine)/graphene oxide modified glassy carbon electrode showed acceptable sensitivity (19.72 µA/µM). The incorporation of Au NPs onto multi-walled carbon nanotube grafted silica network significantly improved the sensitivity towards nanomolar detection of DA (17.8 nA/nM). Employing Au NPs decorated polypyrrole/reduced graphene oxide hybrid sheets as EC sensor exhibited remarkable sensitivity of DA (16.40 µA/µM), the molecularly imprinted oxygen-containing polypyrrole decorated carbon nanotubes composite showed similar sensitivity towards DA (16.18 μA/μM). Using the neural microelectrode array electrodeposited directionally with polypyrrole graphene nanocomposites to detect DA exhibited superior sensitivity (13.933 μA/μM). However, the sensitivity of EC sensors still needs enhancements, and their selectivity should be improved for real-time and in vivo measurements due to interferent biological molecules and biofouling of the sensor surface. To improve the sensing performance of these sensors, utilizing catalysts such as metal and metal oxide nanoparticles and development of other nanomaterials can help to enhance the sensitivity and selectivity. Also, to enhance the spatiotemporal resolution especially in animal models, the miniaturization of the sensing system plays critical role and should be considered. Optical sensing of DA using nanomaterials provides sensitive spectroscopic signals for quick analysis. Plasmonic nanoparticles assembly and disassembly lead to color changes which in turn add features to the colorimetric and spectrophotometric sensing of DA. But these changes occurring in color may result in false positive signals. Employment of smaller nanoparticles could overcome and minimize these undesired results. Fluorescence sensors need accurate modification of the surface and optimization of the chemical conditions to make the interactions between DA and the nanomaterials efficient to obtain the maximal signal intensity. Using nanomaterials in ECL assays has enhanced the signal intensity. The ECL direct detection of DA in the blood using NIR QDs was useful but the sensitivity was poor. In comparison to other detection techniques, the SERS sensors of DA provide higher sensitivity and selectivity. However, expensive equipment is required for analysis, and this remains a challenge. SPR sensors appear as promising technique to detect DA in real time without complex sample preparation steps. Using these simple and ultrasensitive sensors, the measurements for DA concentration can be repeated with good reproducibility. SPR sensing systems do not require separation or labeling of the reagents. In DA detection using these various types of the sensors mentioned, the detection limits in fM range were obtained using the following sensors; ECL (310 fM), PEC (230 fM), SPR (200 fM), PRRS (100 fM), Fluorescence (100 fM), EC (78 fM), and the lowest value was achieved using the SERS sensor (6 fM). Ongoing research in the design and development of carbon and polymers-based nanomaterials to incorporate with SPR technique for high selectivity and sensitivity provides exciting new opportunities for reliable and economic biomedical diagnostic tool for brain disorders related to DA. The real-time continuous detection in vivo and in vitro with high spatiotemporal resolution and long-term stability in the presence of other biological species still be the main objectives of research in the field of DA sensors in the near future.

## Figures and Tables

**Table 1:** Carbon-based electrochemical (EC) sensor for dopamine (DA) detection.

Material	EC Method	LOD	Sensitivity	Linear Range	References
MWCNT-IE	DPV	0.1 µM	8.05 µA/µM	0.5–10 µM	[86]
MWCNT–DHP/GCE	DPV	11 nM	-	50 nM–5 µM	[87]
MWCNT-Nafion/GCE	DPV	2.5 nM	-	10 nM–10 µM	[89]
Sol–gel CCE	AMP SWV	0.07 µM 0.1 µM	0.0414 µA/µM 0.75 µA/µM	0.5–50 µM 0.5–20 µM	[119]
Pd/CNF-CPE	DPV	0.2 µM	-	0.5–160 µM	[120]
CNF-CPE	DPV	0.04 µM	-	0.04–5.6 µM	[121]
(MGNFs)/Si	CV, DPV	0.17 µM	-	1–100 µM	[122]
GC/MWCNTs/Q/Nafion^®^	CV	1.4 µM	121.6 mA/M cm^2^	1.4–300 µM	[91]
GE–CS/GCE	DPV	5 µM	-	5–200 µM	[92]
ITO/MWCNT-g-silica NW/Au NPs	DPV	0.1 nM	17.8 nA/nM	0.1–30 nM	[123]
CNT-AgHCF NPs/GCE	CV	140 nM	-	2.4–130 µM	[124]
graphene	DPV	2.64 µM	-	4–100 µM	[60]
CDP–GS–MWCNTs/GCE	DPV	0.05 µM	-	0.15–21.65 µM	[125]
FGGE	CV, DPV, CA	0.25 µM	-	0.5–50 µM	[126]
graphene-LDH/GCE	CV, SWV	0.3 µM	-	1–199 µM	[127]
NiAl-LDH/G/GCE	CV	9.6 µM	0.022 µA/µM	80–400 µM	[128]
G/Pt/GCE	CV, DPV	30 nM	0.9695 µA/µM	0.03–8.13 µM	[129]
TiO_2_–graphene–GCE	DPV	2 µM	-	5–200 µM	[130]
Cu_2_O/Graphene/GCE	CV	10 nM	-	0.1–10 µM	[131]
MWCNT/GONR/GCE	DPV	80 nM	-	0.5–50 µM	[132]
TiO_2_-GR/4-ABSA/GCE	DPV	0.1 µM	-	1–400 µM	[133]
La/MWCNT	CA	13 nM	-	0.04 µM–0.89 mM	[134]
CPE	CV, DPV	3.7 µM	106.58 µA/mM	8–134 µM	[135]
Graphene	CV	0.5 µM	-	2.5–100 µM	[93]
GS–PTCA/GCE	DPV	0.13 µM	-	0.4–370 µM	[136]
N-dope rGO NG/GCE	DPV	0.25 µM	-	0.5–170 µM	[137]
SPGNE	DPV	0.12 µM	-	0.5–2000 µM	[138]
GNSs	CV, DPV	0.6 µM	-	4–52 µM	[50]
Nafion/graphene/Fc-NH2/GCE	DPV	0.02 µM	-	0.05–200 µM	[139]
TCPP/CCG	DPV	0.01 µM	-	0.01–70 µM	[140]
G-Au NPs/GCE	DPV	1.86 µM	510.2 µA/mM cm^2^	5–1000 µM	[141]
AuNPs-rGOS-ITO	PVD	60 nM	62.7 µA/mM cm^2^	0.02–40 µM	[142]
AuNPs/ERGO/GCE	DPV	0.04 µM	-	0.1–10 µM	[143]
AuNPs–-CD–Graphene	SWV	0.15 µM	-	0.5–150 µM	[144]
sulfonated graphene/GCE	DPV	0.02 µM	-	0.2–20 µM	[145]
{AuNPs/RGO}_20_/GCE	DPV	0.02 µM	-	1–60 µM	[146]
RGO–AuNPs–CSHMs	DPV	0.3 µM	-	1–100 µM	[147]
Pd-RGO/GCE	DPV	100 nM	278 µA/µM cm^2^	2–10 µM	[148]
3D graphene/CNT/Nafion/HRP	CV, AMP	20 nM	470.7 mA/M cm^2^	2–64 µM	[149]
SWCNT–GNS/GCE	DPV	10 nM	-	0.1–52.5 µM	[150]
CuZEA/RGO/GCE	DPV	41 nM	-	0.1–19 µM	[151]
Pd Pt/PDDA–RGO	DPV	0.04 µM	-	4–400 µM	[152]
RGO-Pd/GCE	LSV	0.233 µM	2.62 µA/µM cm^2^	1–150 µM	[153]
NG	CV, LSV	0.93 µM	-	100–450 µM	[154]
HAu-G/GCE	AMP	0.05 µM	-	0.08–600 µM	[155]
NG/PEI/GCE	DPV	0.5 µM	-	1–130 µM	[156]
GO	DPV	0.27 µM	-	1–15 µM	[157]
rGO-P*p*PD/GCE	AMP	0.36 µM	-	5–25 µM 50–200 µM	[158]
Fe_3_O_4_/rGO/GC	DPV	0.08 µM	38.8 A/M cm^2^	0.4–3.5 µM	[96]
CDs–CS/GCE	DPV, CV	11.2 nM	-	0.1–30 µM	[97]
Au@ CDs–CS/GCE	DPV, CV	0.001 µM	-	0.01–100.0 μM	[98]
3D-GF	DPV, CV, AMP	~2 nM	-	0.01–10 µM	[159]
ERGO/HAD/GCE	DPV	19 nM	-	50 nM–400 µM	[160]
IL-G/GCE	DPV	0.812µM	0.063 µA/µM	5–275 µM	[161]
Au NPs@ PS/RGO/GCE	DPV	5 nM	3.44 µA/µM	0.05–20 µM	[162]
Ag NPs/rGO	LSV	5.4 µM	-	10–800 µM	[163]
GN-PSS-Pt	CA	40 nM	302.2 µA/mM cm^2^	0.2 µM–4 mM	[164]
GR–SnO_2_/CILE	DPV	0.13 µM	-	0.5–500 µM	[165]
SDS–GN/SnO_2_	DPV	80 nM	-	0.1–10 µM	[166]
GO/SiO_2_–MIPs	CA	30 nM	-	50 nM–160 µM	[167]
ZnO-sG-Nafion	AMP	1 µM	-	10–800 µM	[168]
MWCNT/GO/GCE	DPV	22 nM	1.53 µA/µM cm^2^	0.2–400 µM	[169]
3D-GN@WO3 NW	AMP	238 nM	1.306 mA/mM cm^2^	10–150 µM	[170]
Au @Pd–RGO/GCE	DPV	2 nM	6.08 µA/µM cm^2^	0.01–100 µM	[171]
Fe_3_O_4_-NH_2_@GS/GCE	DPV	0.126 µM	-	0.2–38 µM	[172]
Pd–RGO	LSV	-	-	2–63 µM	[173]
Pt/RGO/GCE	DPV	0.25 µM	-	10–170 µM	[174]
GNS/PEI/AuNP	DPV	0.2 µM	2.64 µA/µM cm^2^	2–48 µM	[175]
sG/GCE	DPV	2.8 µM	11.67 nA/µM	20 µM–0.4 mM	[176]
ERGO–FA/GCE	AMP	0.19 µM	96.25 µA/µM cm^2^	0.6–1000 µM	[177]
Trp–GR	DPV	0.29 µM	-	0.5–110 µM	[178]
3D–RGO/GCE	DPV	0.17 µM	244.17 mA/mM cm^2^	5 µM–1 mM	[179]
GEF/CFE	DPV	1.36 µM	-	1.36–125.69 µM	[99]
ERGO	AMP, DPV	0.1 µM	-	0.1–10 µM	[180]
ERGO	SWV	20 nM	-	25 nM–5 µM	[181]
(f-RGO)/GCE	DPV	3 µM	-	5–70 µM 100–600 µM	[182]
NiONP–MWCNT–DHP/GCE	DPV	50 nM	1.9 A/M	0.07–4.8 μM	[100]
Pyrolytic carbon	CV, DPV	2.3 µM	0.20 µA/µM cm^2^	18–270 µM	[183]
Ag NPs/SiO_2_/GO/GCE	SWV	0.26 µM	-	2–80 µM	[101]
Au/RGO/GCE	DPV	1.4 µM	-	6.8–41 µM	[102]
CTAB–GO/MWCNT/GCE	DPV	1.5 µM	-	5–500 µM	[62]
ERGO	DPV	0.5 µM	-	0.5–60 µM	[184]
ErGO/CFE	DPV	0.77 μM	-	1.5–224.82 μM	[185]
Au@PPy/RGOS	DPV	18.29 pM	16.4 µA/µM	0.1–5000 nM	[186]
rGO/TiO_2_ {001}/GCE	DPV	6 μM	-	2–60 μM	[187]
Fe_3_O_4_/rGO/GCE	DPV	0.12 μM	2.733 µA/µM	0.5–100 μM	[188]
rGO–CDs/GCE	DPV	1.5 nM	-	10 nM–450 μM	[189]
Lap/G/GCE	DPV	0.25 μM	-	0.5–170 μM	[190]
Cu(tpa)–EGR/GCE	DPV	0.21 μM	-	1–50 μM	[191]
Cr–G/GCE	SWV	-	-	1–10 μM 10–100 μM	[192]
CNPEs	FSCV	25 nM	-	0.1–10	[105]
CNS–Ta	FSCV	8 nM	0.002 nA/µM µm^2^	100 nM–100 μM	[106]
G–AN	DPV	0.24 μM	-	1–100 μM	[107]
rGO/Fe_3_O_4_/GCE	AMP	7 nM	3.15 µA/µM cm^2^	0.01–100.55 μM	[193]
GNB/GCE	CV	0.58 μM	0.95 µA/µM cm^2^	2 μM–0.2 mM	[108]
(PLL/GO/GCE)	DPV	21 nM	19.72 µA/µM cm^2^	0.5–35 μM	[109]
CS/N, GQDs@SPCE	DPV	0.145 μM	418 µA/mM cm^2^	1–100 μM 100–200 μM	[111]
3D pGO–Au NP–pGO–modified ITO	CV	1.28 µM	-	0.1–30 µM	[112]
L–His SNGC	SWV	0.1 µM	-	50–200 µM	[113]
PEDOT: Nafion-SDS PEDOT: Nafion-SDBS	FSCV	12 nM 9 nM	23.7 nA/µM 34 nA/µM	-	[114]
Carbon Nanorod	FSCV	60 nM	5 nA/µM	-	[115]
MWCNT-modified UNCD	DPV	9.5 nM	36 µA/µM cm^2^	33 nM–1 µM	[116]
rGO–Co_3_O_4_/GCE	AMP	0.277 µM	0.389 µA/µM cm^2^	1–30 µM	[69]
rCAG	AMP	30 nM	66.8 μA/mM cm^2^	0.2–90 µM	[70]
f-CNTs/GC	DPV	5.3 µM	-	8–45 µM	[2]
GCE/PANI–NiO GCE/PANI–ZnO GCE/PANI–Fe_3_O_4_	CV	15.3 nM 16.6 nM 17.6 nM	0.078 µA/µM 0.089 µA/µM 0.058 µA/µM	2.4–20 µM	[73]
PEDOT–LSG	DPV	0.33 µM	0.22 µA/µM	1–150 µM	[117]
MnO_2_ NWs/ERGO/GCE	CV, SDLSV	1 nM	-	0.01–0.10 µM 0.10–1.0 µM 1.0 µM–80 µM	[118]

MWCNT-IE—MWCNT-intercalated graphite electrodes; DHP—Dihexadecylphosphate; GCE—Glassy carbon electrode; CNF—Carbon nanofibers; MGNFs—Multilayer graphene nanoflake films; g-silica NW—Grafted silica network; AgHCF—Silver hexacyanoferrate; CDP—Polycyclodextrin; GS—Graphene sheet; FGGE—Functionalized-graphene modified graphite electrode; LDH—Layered double hydroxide; GONR—Graphene oxide nanoribbon;4-ABSA—4-aminobenzenesulfonic acid; La—Lanthanum; PTCA—3,4,9,10-perylenetetracarboxylic acid; NG—Nitrogen doped graphene; SPGNE—Screen-printed graphene electrode; GNSs—Graphene nano-sheets; Fc-NH_2_—1-[(4-amino) phenylethynyl] ferrocene; TCPP—Meso-tetra (4-carboxyphenyl) porphine; CCG—Chemically converted graphene; rGOS—Reduced graphene oxide sheets; CSHMs—Chitosan/silica sol–gel hybrid membranes; HRP—Horseradish peroxidase; CuZEA—Cu-zeolite A; HAu-G—Hollow gold-graphene; P*p*PD—Poly(*p*-phenylenediamine); 3D-GF—3D graphene foam; HAD—1,6-hexanediamine; PS—Polystyrene; GN-PSS—Graphene- Poly (sodium 4-styrenesulfonate); CILE—Carbon ionic liquid electrode; MIPs—Molecularly imprinted polymers; sG—Solar graphene; 3D-GN—Three-dimensional graphene network; WO_3_—Tungsten trioxide; NW—Nanowire; FA—ferulic acid; Trp-GR—Tryptophan-functionalized graphene nanocomposite; f-RGO—Flower-like graphene-nanosheet clusters; Lap—Laponite; Cu(tpa)—Copper terephthalate metal-organic framework.

**Table 2:** Polymer-based EC sensor for DA sensing.

Material	EC Method	LOD	Sensitivity	Linear Range	References
PPy (DS)/GCE	CV, CA	40 nM	-	0.1–10 µM	[201]
poly (2-picolinic acid)/GCE	CV	30 nM	-	0.25–10 mol/dm^3^	[203]
(PNAANI)/GCE	DPV	16.8 nM	-	0.5–20 µM	[204]
TPY-modified PFE/GC and graphite/polyester composite electrodes	DPV	0.2 µM	62 nA/µM	1–250 µM	[205]
OPPD-CFME	SWV	10 nM	2.6 nA/µM	50 nM–10 µM	[206]
P3MT/γ-CD in tetrabutylammonium hexafluorophosphate/acetonitrile solution	SWV	0.2 µM	-	0.5–50 µM	[228]
PPy/FCNMCPE	LSV DPV	38.6 µM 15.1 µM	-	0.10–1.20 mM 0.20–0.95 mM	[208]
Nafion/Cu-MPS/GCE	SWV	0.05 µM	-	0.08–5 µM	[209]
Nafion/SWNTs/P3MT	DPV	5 nM	-	0.020–0.10 µM 0.10–1.0 µM 1.0–6.0 µM	[229]
PEDOT/GCE	SWV	-	-	0.1–0.5 mM	[194]
F3GA/PDAN/GCE	LSV	0.1 µM	0.036 µA/µM	5–100 µM	[212]
tyrosinase–SWNTs–PPy	AMP	5 µM	467 mA/Mcm^2^	5–850 µM	[230]
Nano-Cu/PPy/GCE	DPV	0.85 nM	-	1 nM–0.1 µM	[231]
Pt/P3MT/Pd	DPV	8 nM	1.44 µA/µM	0.05–1 µM	[232]
TS-PANI/GCE	AMP	0.7 µM	28.36 µA/mM	0.01–0.3 mM	[233]
LbL deposited PANI–AuNP	DPV	3 µM	10.1 µA/mM	7–148 µM	[216]
EDTA-RG/Nafion/GCE	DPV	0.01 µM	-	0.2–25 µM	[234]
PNMPy/PS	CV	1.5 µM	-	10–20 mM	[235]
GSCR-MIPs/GCE	CA	0.1 µM	-	0.1 µM–0.8 mM	[236]
PPy/ERGO	DPV	23 nM	-	0.1–150 µM	[237]
PMR/CPE	CV	5 nM	-	10 nM–0.1 µM 0.1 µM–1 mM	[238]
PEDOT/Pt electrode in presence of SDS	CV	61 nM 86 nM	-	0.5–25 µM 30–100 µM	[239]
Lac/PPy/MWCNT/Pt	DPV	0.14 µM	-	0.5–4.75 µM	[240]
MIPs/MWCNTs/GCE	DPV	60 nM	-	0.63–100 µM	[241]
OPPy–MSA–MWCNTs/Au electrode	DPV	0.4 nM	7.61 µA/µM	1 nM–2.87 µM	[242]
PVP/GR/GCE	AMP	0.2 nM	-	0.5 nM–1.13 mM	[243]
Aptamer/GR–PANI/GCE	SWV	1.98 pM	-	0.007–90 nM	[244]
AuNPs @PANI	DPV	5 µM	-	10–1700 µM	[215]
PEDOT-Au_nano_/Au in presence of SDS	LSV	0.39 nM 1.55 nM	0.0381 µA/µM	0.5–20 µM 25–140 µM	[218]
Pty/GCE	LSSV	142 nM	-	1–7 µM	[245]
RGO-HDPPy/GCE	DPV	0.3 nM	-	1–8000 nM	[246]
PAPT/GCE	DPV	0.2 µM	-	0.95–380 µM	[247]
(Gr/CS)_5_/GCE	DPV	0.05 µM	-	0.1–140 µM	[248]
(MPEG)/GCE	DPV	46.8 nM	198.4 µA/mM	1–140 µM	[249]
polyDAN-RB4/GCE	DPV	0.061 µM	-	0.1–150 µM	[219]
PEDOT/CNT/CPE	DPV	20 nM	-	0.1–20 µM	[220]
P-4-ABA/GCE	DPV	1 µM	-	5–100 µM	[250]
Nafion-CNT- ABTS/ITO	DPV	1.75 µM	1.334 µA/µM	1.87–20 µM	[251]
GO-PEDOT/GCE	CV	83 nM	0.151 µA/µM	1–40 µM	[252]
PEDOT/RGO/GCE	AMP	39 nM	-	0.1–175 µM	[253]
GR–CS/CPE	DPV	98.2 nM	-	0.1 mM–0.2µM	[254]
ERGO-P/GCE	DPV	35 nM	-	1–500 µM	[255]
PEDOT: PSS/ITO	AMP	1 µM	196 mA/M cm^2^	0.01–0.9 µM	[223]
PPy/CNTs-MIPs/GCE	DPV	10 pM	16.18 μA/μM	50 pM–5 μM	[256]
PPyox-PTSA/Ag-NP/Pt	DPV	0.58 nM	-	1–120 nM	[257]
(PoPD)/ERGO/GCE	DPV	7.5 µM	-	10–400 µM 400–800 µM	[258]
AuNP/PANI	AMP	0.91 µM	0.09284 μA/μM	1 µM–0.1 mM	[259]
MBIP	DPV	6 nM	-	0.02–7 μM	[260]
PPy/graphene/nMEA	AMP	4 nM	13933.12 μA/mM cm^2^	0.8–10 μM	[261]
PEDOT/rGO/aptamer	DPV	78 fM	-	1 pM–160 nM	[262]
GO/AuNPs/pDAN-EDTA	AMP	5 nM	-	10 nM–1 µM	[224]
PILs/PPy/GO	DPV	73.3 nM	2.499 μA/μM	4–18 µM	[263]
(Pt/UltraPPy–GCE)	DPV	0.67 nM	-	0.01–400 µM	[264]
Au NPs/OPPy nanotube arrays electrode	SWV	10 nM	-	25 nM–2.5 µM	[265]
Au/PEDOT–Pt–Ag/AgCl	DPV	0.1 µM	1.65 μA/μM cm^2^	0.2– 300 µM	[266]
PEDOT/DNA/CPE	AMP	74 nM	-	0.25–66.5 µM	[267]
PEDOT/IL/GCE	AMP	51 nM	-	0.2–312 µM	[268]
AuNPs-PTAP/GCE	DPV	0.017 µM	8.170 μA/μM	0.15–1.5 µM	[269]
PEDOT/DES/GC	DPV	1.3 µM	1.5 mA/mM cm^2^	5–180 µM	[270]
ET-SDBS-NPPy/ERGO	SWV	20 nM	13.07 μA/μM	0.1–100 µM	[271]
POA/CNTs/GCE	DPV	0.12 µM	8.71 μA/μM	10.0–260.0 µM	[272]
MIP/PPy NWs/GCE	DPV	33 nM	-	50 nM–100 µM	[273]
PEDOT: PSS/ITO	DPV	6.84 µM	826 nA/μM cm^2^	1–50 µM	[274]
GR/pAHWSA/SPCE	SWV	2 nM	-	0.05–100 µM	[226]
PEDOT/GO/CFE	CV	0.22 µM	-	0.5–10 µM	[225]
PTh/GPE	LSV	1 µM	-	10–180 µM	[275]
PPy/graphene/GCE	CV	2.3 µM	363 μA/mM cm^2^	0.1–1 mM	[276]
pHQ/AuNPs/NF	DPV	41.9 nM	6.663 μA/μM	0.1–10 µM	[277]

GCFME—Glassy carbon fibre microelectrodes; PNAANI—Poly(N-acetylaniline); TPY—2,4,6-triphenylpyrylium ion stabilized inside zeolite matrix; P3MT/γ-CD—Poly-3-methylthiophene combined with γ -cyclodextrin; P3MT—Poly-3-methylthiophene; TS-PANI—Tetragonal star like Polyaniline; LbL—Layer-by-layer; EDTA—Ethylenediamine triacetic acid; PNMPy—Poly(N-methylpyrrole); PS—Polystyrene; SCR—Graphene sheets/Congo red; PMR—Poly (methyl red); Lac—Laccase; MSA—Mercaptosuccinic acid; PVP—Polyvinylpyrrolidone; Pty—Polytyramine; HDPPy—Highly dispersed polypyrrole nanospheres; PAPT—Poly(2-amino-5-(4-pyridinyl)-1,3,4-thiadiazole); MPEG—ethoxypolyethylene glycol; polyDAN-RB4—Reactive blue-4 (RB4) dye entrapped poly1,5-diaminonaphthalne (polyDAN); P-4-ABA—Poly (4-aminobutyric acid); ABTS—An 2,2′-azino-bis (3-ethylbenzthiazoline-6-sulfonic acid); PPyox-PTSA—P-toluene sulfonic acid-doped ultrathin polypyrrole; PoPD—o-phenylenediamine; MBIP—Molecularly bioimprinted polymer; nMEA—Neural microelectrode array; pDAN-EDTA—Ethylenediamine triacetic acid immobilized-poly (1,5-diaminonaphthalne); PILs—Poly (ionic liquids); PTAP—Poly (2,4,6- triaminopyrmidine); DES—Deep eutectic solvents; ET-SDBS-NPPy—Electrochemically treated sodium dodecyl benzene sulfonate doped nano polypyrrole; POA—Poly(o-anisidine); pAHWSA—Poly 4-amino-3-hydroxy- 1-naphthalenesulfonic acid; PTh—Polythiophene; pHQ—Poly(hydroquinone); NF—Nickel foam.

**Table 3:** DA optical sensors.

Type of Sensor	Determination Method	LOD	Linear Range	References
Luminol–H_2_O_2_–Au NPs	CL	0.19 nM	0.001–5 µM	[333]
MPA-modified CdTe QDs	ECL	50 nM	0.05–5 µM	[334]
DA–RC	SPR	0.085 ng/mL	0.085–700 ng/mL	[332]
CdSe QDs	ECL	500 nM	0.5–70 µM	[335]
CdSe–ZnS QDs-GSH/ATTO−590	Fluorescence	1 µM	-	[336]
Potassium Ferricyanide-Fe (III)	Spectrophotometry	0.045 µg/mL	0.05–6.00 µg/mL	[337]
Luminol–H_2_O_2_–ZnO NPs	CL	5 nM	0.005–6.5 µM	[338]
AuNPs–Cu^2+^	Spectrophotometry	30 nM	33 nM–0.1 µM 0.3–4.5 µM	[339]
Ag NPs	Spectrophotometry	1.2 µM	3.2–20 µM	[340]
PVP/Ag NPs	Spectrophotometry	0.8 µM	3.2–20 µM	[278]
CdTe QDs/CNTs/CS/ITO	ECL	24 pM	50 pM–10 nM	[314]
CdSe/ZnS QDs/GCE	ECL	50 nM	0.1–20 µM	[341]
Au NCs/ITO	ECL	-	2.5–47.5 µM	[342]
GO	Fluorescence	94 nM	0.25–20 µM	[343]
Au NPs-BDA	Colorimetry	0.36 µM	0.54–5.4 µM	[344]
DA–MBA–DSP–AuNPs	Spectrophotometry	0.5 nM	5–180 nM	[345]
Ag NPs	Spectrophotometry	60 nM	0–0.6 µM	[346]
DNA mediated Ag nanostructure	Fluorescence	6 nM	0–200 nM	[347]
β–CD/MSN	Fluorescence	50 nM	50 nM–20 µM	[348]
MIP–Au electrode	SPR	1 pM	-	[291]
Au NPs	SERS	1 ng/mL	1 nM–1 mM	[307]
AgNPs-Fe (NTA)	SERS	60 pM	0.5–4 nM	[349]
ZnSa NWs–Ag NPs	Fluorescence	3 nM	0–300 nM	[350]
CdS spherical aggregates	PL	10 nM	0–30 µM	[7]
CDs@MIP	Fluorescence	1.7 nM	25–500 nM	[351]
CdS QDs/ITO	ECL	-	1 μM–10 mM	[352]
Ag_2_Se QDs/PEI/MWCNTs/GCE	ECL	100 nM	0.5–19 µM	[353]
RGO–Nafion/Ru NWs/GCE	ECL	0.31 pM	1 pM–10 µM	[354]
CdS-PAMAM/Au NPs	ECL	12 nM	0.05–10 µM	[355]
AuNPs–Cu^2+^	Colorimetry	0.2 µM	0.5–10 µM	[303]
DSP–AuNPs + Fe^3+^	Colorimetry	2 nM	5–600 nM	[356]
DMAP–AuNP	Colorimetry	5 nM	10–100 nM	[357]
Tb^3+/^AgNPs	Fluorescence	0.42 nM	2.4–140 nM	[358]
Fe_3_[Fe (CN)_6_]_2_	RRS	3.43 ng/mL	0.06–1 µg/mL	[359]
Formaldehyde–KMnO4	CL	10 nM	0.031–17 µM	[48]
Calcein blue–Fe^2+^	Fluorescence	10 μM	50 μM–1 mM	[360]
BCG/Sephadex LH-20 gel	SPS	1.7 µM	0.4–1.6 µg/mL	[361]
TGA-capped CdTe QD –Lac	PL	0.16 µM	0.3–100 µM	[362]
BSA-Au NCs	Fluorescence Colorimetry	10 nM	10 nM–1 µM	[363]
Ag NCs @APTES–GD/ITO	ECL	0.92 nM	8.3 nM–0.83 µM	[364]
Nafion/TiO_2_/GCE	ECL	10 pM	10 pM–0.6 µM	[316]
CNTs/DSP–QDs/GCE	ECL	26 pM	50 pM–10 nM	[365]
F-CuInS_2_ QDs	Fluorescence	0.2 µM	0.5–40 µM	[366]
CdTe QDs @silica	Fluorescence	0.241 µM	0.5 µM–0.1 mM	[367]
CNPs/Fe^3+^	Fluorescence	68 nM	0.1–10 µM	[368]
AuNPs–AHMT	Colorimetry	70 nM	0.20–1.10 µM	[369]
DTSSP–AuNPs	Colorimetry	10 nM	0.02–0.80 µM	[306]
AuNRs–Ag^+^	Colorimetry	47 nM	0.20–12 µM	[370]
CS-Au nanoshell	SERS	-	1–10 mM	[308]
Ag NPs-PMA	Spectrophotometry	0.527 µM	0.527–15.8 µM	[371]
Zr [Fe (CN)6] NPs	RRS	0.392 ng/mL	0.03–1.3 mg/mL	[372]
Au NP–RGO–K_2_S_2_O_8_	ECL	6.2 nM	0.02–40 µM	[373]
APTES-capped ZnO QDs	Fluorescence	12 nM	0.05–10 µM	[374]
MA/AuNPs	Colorimetry	30 nM	0.3 µM–10 mM	[302]
MA/AuNPs	Colorimetry	33 nM	33 nM–3.33 mM	[375]
Carbon dots (CDs)	Fluorescence	33 µM	33–1250 µM	[376]
CdSe/ZnS QDs/A	Fluorescence	29.3 nM	100 nM–20 µM	[53]
AgQL	Fluorescence	16 nM	0–300 nM	[377]
PDA NPs	Fluorescence	40 nM	0.1–20 µM	[378]
CdSe QDs– ABA/GCE	ECL	3 nM	10 nM–3 µM	[379]
rGO/MWCNTs/AuNPs/GCE	ECL	67 nM	0.20–70 µM	[380]
Ru(bpy) _3_^+2^/ordered mesoporous carbon/Nafion/GCE	ECL	1.7 nM	5 nM–500 µM	[381]
CS-Au nanocomposite	SERS	1 mM	1–10 mM	[309]
(GT)_15_ DNA–and (GU)_15_ RNA-wrapped SWCNTs	Fluorescence	11 nM	10 nM–10 µM	[280]
Porous Ag paper electrode and silica CDs	ECL	4.3 mU/mL	0.01–50 U/mL	[382]
(AuNF@g–C_3_N_4_–PANI)	ECL	1.7 nM	5 nM–1.6 µM	[383]
(BQ) + CdS QDs	PEC	8 nM 0.1 nM	20 nM–50 μM 2 nM–10 μM	[384]
J–Aggregate Nanotubes	Spectrophotometry	0.4 nM	0–100 nM	[385]
Ce (IV)-Na_2_S_2_O_3_-C-dot	CL	1 nM	2.5 nM–20 μM	[386]
(CDs/TYR)	Fluorescence	60 nM	0.206–131.8 µM	[387]
BSA–AuNC–Cu^2+^	Fluorescence	0.01 µM	0–3.5 µM	[388]
DA antibodies/Au NPs/ITO	SPR	1 nM	0.001–100 µM	[294]
GQDs	Fluorescence	0.008 µM	0–60 µM	[389]
TGA–CdS QDs	Fluorescence	2.55 nM	0.394 µM–46.7 nM	[390]
L–Cys–capped InP/ZnS QDs	Fluorescence	875 pM	800 pM–100 nM	[391]
PPy/GQDs	Fluorescence	10 pM	5–8000 nM	[392]
ds–DNA templated Cu NPs	Fluorescence	20 pM	0.0001–10 µM	[393]
rGO/Ag Nanotriangle	SERS Fluorescence Absorption	1.2 µM 25 µM 12 µM	2.5–500 µM 50–500 µM 25–500 µM	[394]
Si NPs	Fluorescence	0.3 nM	0.005–10 µM	[395]
(GQDs –TiO_2_) nanocomposites/GCE	PEC	6.7 nM	0.02–105 µM	[396]
HCNTs–PAH–CdSe QDs	ECL	0.2 nM	1 nM–20 µM	[397]
g–C_3_N_4_ NSs–TCA	ECL	2.4 pM	6 pM–30 nM	[315]
GCE/PTh–D@NH_2_-G(graphene)/Nafion	ECL	0.04 μM	0.1–50.0 μM	[398]
GCE/AuNPs/L–Cys–C60–APBA	ECL	0.003 µM	0.01 µM–40 µM	[399]
luminol–H_2_O_2_–HKUST-1	CL	2.3 nM	0.1–0.70 µM	[400]
CdSeTe/ZnS core–shell QD-CS films/GCE	ECL	100 nM	3.75–450 µM	[401]
TiO_2_/Pt electrode	ECL	2.7 pM	4 pM–18 nM	[317]
MA/AuNPs	Colorimetry	33 nM	33 nM–3.33 mM	[302]
FB-AuNPs/NsNHS-AuNPs	Absorption Fluorescence	1.2 nM 2.9 nM	5–100 nM	[54]
DA capped Au NPs modified with (TGA^2−^)	Colorimetry	94 nM (serum)	0–1 µM	[402]
Au NPs	PRRS	0.1 pM	1 pM–1 µM	[403]
CuS–rGO	Spectrophotometry	0.48 µM	2–100 µM	[404]
Au @ Ag NR dimers based on aptamers	SERS	0.006 pM	0.01–10 pM	[405]
Ag NPs/MIL–101 (Fe)	SERS	0.32 pM	1.054 pM–210.8 nM	[406]
(GSH) protected (Au NCs)	Fluorescence	1 nM	1 nM–1 mM	[407]
NH2-β-CD-Au NCs	Fluorescence	2 nM	5–1000 nM	[408]
PFPBA NPs	Fluorescence	38.8 nM	0.025–10 µM	[409]
Ag	SERS	8.3 nM	0.1–50 µM	[410]
Ag@GO	SPR	30 nM	100 nM–2 µM	[411]
GQDs	Fluorescence	8.2 nM	0.01–50 µM	[412]
GQDs	Fluorescence	0.09 µM	0.25–50 µM	[413]
Aptamer + AuNPs	ColorimetryFluorescence	0.14 μM 78.7 nM	0.17–4.0 μM 0.083–2.0 μM	[304]
B-N-CDs	Fluorescence	0.1 pM	1 pM–1 μM	[313]
Aptamer + Ru complex-QDs	Fluorescence	19 nM	0.03–0.21 μM	[414]
CdSe/ZnS QDs	Fluorescence	100 nM	-	[415]
Ag NPs	SPR	0.2 µM	0.2–30 µM	[416]
Ag NPRs	Colorimetry	0.16 nM	0.5–100 nM	[417]
aptamer–CDs–NG	Fluorescence	0.055 nM	0.10–5.00 nM	[418]
TiNTs–ITO and PB-Pt	ECL	30 nM	0.1–5 μM	[419]
Ag NPs	Colorimetry	6.13 μM	1–500 µM	[305]
β-CD–Au NPs	Colorimetry	30 nM	20–250 nM 350–1600 nM	[420]
PIMH iron (III) into PBVC NF	Spectrophotometry	6.4 mg/L	0.148–184 mg/L	[421]
Poly (DA)@GQDs	Fluorescence	80 nM	1–200 µM	[422]
GQDs	Fluorescence	0.022 µM	1–200 µM	[423]
CNDs	Fluorescence	47 pM	0–20 µM	[424]
NanoMoS_2_/Gold Electrode	PEC	2.3 pM	10 pM–10 µM	[425]
Ag@ Au core-shell	Colorimetry	5 µg/mL	1–30 µg/mL	[426]
Pt	SPR	50 pM	0.1 nM–32 µM	[427]
DAAPT-AuNPs	SPR	200 fM	100 µM–2 mM 200 fM–20 nM	[31]
Molecular Imprinted GNP/SnO_2_	SPR	31 nM	0–100 µM	[428]
MoS_2_ QDNS	Fluorescence	0.9 nM	2.5 nM–5 µM 5 µM–10.4 µM	[429]
S-CDs@AuNPs	Colorimetry	0.23 µM	0.81–16.80 µM	[430]
Au/graphene/DBA D-POF	SPR	-	0.1 nM–1 µM	[431]

MPA—Mercapopropionic acid; ECL—Electrochemiluminescence; GSH—Glutathione; PVP—Polyvinylpyrrolidone; DSP—3,3′-Dithiodipropionic acid di (*N*-hydroxysuccinimide ester); β-CD—β -cyclodextrin; MSN—Mesoporous silica Nanoparticles; Fe (NTA) —Iron-nitrilotriacetic acid; ZnSa—Zinc–salophen; PAMAM—Polyamidoamine; DMAP—4-(dimethylamino) pyridine; BCG—Bromocresol green; TGA—Thioglycolic acid; BSA—Bovine serum albumin; APTES—3-aminopropyl-triethoxysilane; F-CuInS2—3-aminophenyl boronic acid-functionalized CuInS_2_; AHMT—4-amino-3-hydrazino-5-mercapto-1,2,4-triazol; DTSSP—Dithiobis(sulfosuccinimidylpropionate); AuNRs–Ag^+^—Au core–Ag shell nanorods; PMA—Polymethacrylate; Zr[Fe(CN)6]—Zirconium hexacyanoferrate (II); AgQL—Semiquinone form after oxidization of L in the presence of Ag(I); PDA—Polydopamine; ABA—p-Aminobenzoic acid; AuNF@g-C_3_N_4_–PANI—Gold nanoflower @graphitic carbon nitride polymer nanosheet–polyaniline hybrids; BQ—Benzoquinone; CDs/TYR—Carbon dots/tyrosinase; HCNTsPAH-CdSe QDs—Helical CNTs, polyallylamine hydrochloride and CdSe QDs; g-C_3_N_4_ NSs–PTCA—Graphite-like carbon nitride nanosheets/3,4,9,10-perylenetetracarboxylic acid hybrids; PTh-D—Poly[3-(1,1′-dimethyl-4-piperidinemethylene) thiophene-2,5-diyl chloride]; APBA—3-aminophenyl-boronic acid; HKUST-1—(Hong Kong University of Science and echnology): MOF; copper nodes with 1,3,5-benzenetricarboxylic acid struts between them; FB-Au NPs—Fluorescein modified gold nanoparticles; NsNHS-AuNPs—Nile blue modified gold nanoparticles; PRRS—Plasmonic resonance Rayleigh scattering; MIL-101 (Fe) —A typical metal organic framework; Ag NPRs—Silver nanoprisms; TiNTs—Titania nanotubes; PB—Platinum black; PIMH—Tris-(2,2′-pyridylimidazole); PBVC—Poly (vinylbenzyl chloride);CNDs—Carbon nanodots; S-CDs—S-doped carbon dots.

## Abbreviations

3D CAG	Three- dimensional carbon aerogel electrode
3D-GF	3D graphene foam
3D-GN	Three-dimensional graphene network
4-ABSA	4-aminobenzenesulfonic acid
5-HT	5-hydroxytryptamine
AA	Ascorbic acid
ABA	p-Aminobenzoic acid
ABTS	An 2,2′-azino-bis (3-ethylbenzthiazoline-6-sulfonic acid)
Ag NPRs	Silver nanoprisms
Ag NPs	Silver nanoparticles
Ag NT	Silver nanotriangle
AgHCF NPs	Silver hexacyanoferrate NPs
AgQL	Semiquinone form after oxidization of L in the presence of Ag(I)
AHMT	4-amino-3-hydrazino-5-mercapto-1,2,4-triazol
AMP	Amperometry
AN	Acupuncture needle
AP	Acetaminophen
APBA	3-aminophenyl-boronic acid
APTES	3-aminopropyl-triethoxysilane
ATR	Attenuated total reflection
Au NCs	Au Nanoclusters
Au NPs	Gold Nanoparticles
AuNF@g-C_3_N_4_–PANI	Gold nanoflower @graphitic carbon nitride polymer nanosheet–polyaniline hybrids
Au NPs @PS	Gold nanoparticles coated polystyrene
Au NPs–CD–Gra	Graphene decorated with gold nanoparticles
AuNRs–Ag^+^	Au core–Ag shell nanorods
BCG	Bromocresol green
B-N-CDs	Carbon dots with boronic acid and amino groups
BQ	Benzoquinone
BSA	Bovine serum albumin
CA	Chronoamperometric
CCE	Carbon composite electrode
CCG	Chemically converted graphene
CD	Cyclodextrin
CDP	Polycyclodextrin
CDs	Carbon dots
CDs/TYR	Carbon dots/tyrosinase
CF	Carbon fiber
CFE	Carbon fiber electrode
CFMEs	Carbon-fiber microelectrodes
CILE	Carbon ionic liquid electrode
CL	Chemiluminescence
CNF	Carbon nanofibers
CNPEs	Carbon nanopipette electrodes
CNS	Carbon nanospikes
CNTs	Carbon nanotubes
CNTy-D	CNT yarn disk-shaped
CNTYMEs	Carbon nanotube yarn microelectrodes
CPE	Carbon paste electrode
C–PVC	Carbon–polyvinylchloride
CS	Chitosan
CSHMs	Chitosan/silica sol–gel hybrid membranes
CTAB	Hexadecyl trimethyl ammonium bromide
Cu(tpa)	Copper terephthalate metal-organic framework
Cu–Mo	Copper–molybdenum
Cu-MPS	Copper-(3-mercaptopropyl) trimethoxy silane
CuZEA	Cu-zeolite A
CVD	Chemical vapor deposition
DA	Dopamine
DAAPT	Dopamine DNA aptamer
DA-RC	D_3_ dopamine receptor
DBA	Dopamine-binding aptamer
DES	Deep eutectic solvents
DHP	Dihexadecylphosphate
DMAP	4-(dimethylamino) pyridine
DOPA	3-(3,4 dihydroxyphenyl)-alanine
DOPAC	3,4-Dihydroxyphenyl acetic acid
D-POF	D-shaped plastic optical fiber
DPV	Differential pulse voltammetry
DS	Dodecyl sulfate
ds-DNA	Double stranded DNA
DSP	3,3′-Dithiodipropionic acid di(*N*-hydroxysuccinimide ester)
DTSSP	Dithiobis(sulfosuccinimidylpropionate)
EC	Electrochemical
ECL	Electrochemiluminescence
EDOT	3,4-ethylenedioxythiophene
EDTA	N-(trimethoxysilylpropyl) ethylenediamine triacetic acid
EP	Epinephrine
EPD	Electrophoretic deposition
ERGO	Electrochemically reduced graphene oxide
ERGO–FA	Electrochemically reduced graphene oxide–ferulic acid
ET-SDBS-NPPy	Electrochemically treated sodium dodecyl benzene sulfonate doped nano polypyrrole
F3GA	Cibacron Blue
FB-Au NPs	Fluorescein modified gold nanoparticles
FCN	Ferrocyanide
Fc-NH_2_	1-[(4-amino) phenylethynyl] ferrocene
FCNMCPE	Ferrocyanide modified carbon paste electrodes
f-CNTs	Fiber-like carbon nanotubes
F-CuInS2 QDs	3-aminophenyl boronic acid-functionalized CuInS_2_ QDs
Fe (NTA)	Iron-nitrilotriacetic acid
FET	Field-effect transistor
FGGE	Functionalized-graphene modified graphite electrode
f-MWCNTs	Functionalized multi-walled carbon nanotubes
f-RGO	Flower-like graphene-nanosheet clusters
FSCV	Fast-scan cyclic voltammetry
FTIR	Fourier transform infrared
GABA	Gamma-aminobutyric acid
G-AN	Graphene-modified acupuncture needle
g-C_3_N_4_ NSs–PTCA	Graphite-like carbon nitride nanosheets/3,4,9,10-perylenetetracarboxylic acid hybrids
GCE	Glassy carbon electrode
GCPE	Glassy carbon paste electrode
GD	Glutaric dialdehyde
GE	Graphite electrode
GEF	Graphene flowers
GNBs	Graphene nanobelts
GN-PSS	Graphene- Poly (sodium 4-styrenesulfonate)
GNSs	Graphene nano-sheets
GO	Graphene oxide
GONRs	Graphene oxide nanoribbons
GPE	Graphite paste electrode
GS	Graphene sheet
GSCR	Graphene sheets/Congo red
GSH	Glutathione
g-silica NW	Grafted silica network
HAu-G	Hollow gold-graphene
HCNTsPAH-CdSe QDs	Helical CNTs, polyallylamine hydrochloride and CdSe QDs
HDA	1,6-hexanediamine
HDPPy	Highly dispersed polypyrrole nanospheres
HKUST-1	(Hong Kong University of Science and echnology):MOF;copper nodes with 1,3,5-benzenetricarboxylic acid struts between them
HPLC	High performance liquid chromatography
HRP	Horseradish peroxidase
IL	Ionic Liquid
ITO	Indium tin oxide
L	N,N′-Bis(salicylidene)-1,3-propanediamine
La	Lanthanum
Lac	Laccase
Lap	Laponite
LbL	Layer-by-layer
L-Cys-capped InP/ZnS QDs	L- Cysteine-capped indium phosphide/zinc sulfide QDs
LDH	Layered double hydroxide
L-His	L-Histidine
LOD	Limit of detection
LSG	Laser scribed graphene
LSPR	Localized surface plasmon resonance
LSPs	Localized surface plasmons
LSSV	Linear square stripping voltammetry
LSV	Linear sweep voltammetry
MA	Melamine
MBA	4-mercaptophenylboronic acid
MBIP	Molecularly bioimprinted polymer
MGNFs	Multilayer graphene nanoflake films
MIL-101 (Fe)	A typical metal organic framework
MIP	Molecularly imprinted polymer
MOFs	Metal-organic frameworks
MoS_2_ QDNS	MoS_2_ quantum dots dispersed over MoS_2_ nanosheets
MPEG	Methoxypolyethylene glycol
MSA	Mercaptosuccinic acid
MSN	Mesoporous silica Nanoparticles
MWCNT-IE	Multi-walled carbon nanotubes intercalated graphite electrodes
MWCNTs	Multi-walled carbon nanotubes
N, GQDs	Nitrogen-doped graphene quantum dots
Nb	Niobium
NF	Nickel foam
NG	Nitrogen-doped graphene
NH_2_-β-CD	Mono-6-amino-β-cyclodextrin
NiONPs	Nickel oxide nanoparticles
nMEA	Neural microelectrode array
NsNHS-AuNPs	Nile blue modified gold nanoparticles
NTs	Neurotransmitters
NW	Nanowire
OPD	o-phenylenediamine
OPPD	Poly-(1,2-phenylenediamine)
OPPy	Overoxidized Polypyrrole
P	5,15-pentafluorophenyl-10,20-*p-*aminophenylporphyrin
P3MT	Poly-3-methylthiophene
P3MT/γ-CD	Poly-3-methylthiophene combined with γ -cyclodextrin
P-4-ABA	Poly (4-aminobutyric acid)
pAHWSA	Poly 4-amino-3-hydroxy- 1-naphthalenesulfonic acid
PAMAM	Polyamidoamine
PANI	Polyaniline
PAPT	Poly(2-amino-5-(4-pyridinyl)-1,3,4-thiadiazole)
PB	Platinum black
PBA	4-carboxyphenyl-boronic acid
PBS	Phosphate-buffered saline
PBVC	Poly (vinylbenzyl chloride)
Pd	Palladium
PD	Parkinson’s disease
PDA	Polydopamine
PDAN	Poly-1,5-diaminonaphthalene
PDAN-EDTA	Ethylenediaminetetraacetic acid immobilized-poly (1,5-diaminonaphthalene)
PDDA	Poly(diallyldimethylammonium chloride)
PEDOT	Poly(3,4-ethylenedioxythiophene)
PEDOT/IL	PEDOT doped with pure insoluble ionic liquid (IL), 1-ethyl-3-methylimidazolium bis(trifluoromethylsulfonyl)imide
PEI	Polyethylenimine
PFEs	Polymer film electrodes
pGO	Porous graphene oxide
pHQ	Poly(hydroquinone)
PILs	Poly (ionic liquids)
PIMH	Tris-(2,2′-pyridylimidazole)
PL	Photoluminescence
PLL	poly(L-lysine)
PMA	Polymethacrylate
PMB	Poly (methylene blue)
PMR	Poly (methyl red)
PNAANI	Poly(N-acetylaniline)
PNMPy	Poly(N-methylpyrrole)
PNR	Poly (neutral red)
POA	Poly(o-anisidine)
polyDAN-RB4	Reactive blue-4 (RB4) dye entrapped poly1,5-diaminonaphthalne (polyDAN)
PoPD	o-phenylenediamine
P*p*PD	Poly(*p*-phenylenediamine)
PPy	Polypyrrole
PPyox-PTSA	P-toluene sulfonic acid-doped ultrathin polypyrrole
PRRS	Plasmonic resonance Rayleigh scattering
PS	Polystyrene
PSPs	Propagating surface plasmons
PSS	Poly (sodium 4-styrenesulfonate)
PTAP	Poly (2,4,6- triaminopyrmidine)
PTCA	3,4,9,10-perylenetetracarboxylic acid
PTh	Polythiophene
PTh-D	Poly[3-(1,1′-dimethyl-4-piperidinemethylene) thiophene-2,5-diyl chloride]
PTSA	P-toluene sulfonic acid
Pty	Polytyramine
PVP	Polyvinylpyrrolidone
Q	Quercetin
QDs	Quantum dots
rGO	Reduced graphene oxide
rGOS	Reduced graphene oxide sheets
RI	Refractive index
RRS	Resonance Rayleigh Scattering
S-CD	S-doped carbon dots
SDBS	Sodium dodecyl benzene sulfonate
SDLSV	Second-order derivative linear sweep voltammetry
SDS	Sodium dodecyl sulfate
SE	Serotonin
sG	Solar graphene
SiO_2_	Mesoporous silica
SNGC	Sonogel-Carbon electrode
SPCE	Screen-printed carbon electrode
SPE	Screen-printed electrode
SPGNE	Screen-printed graphene electrode
SPR	Surface plasmon resonance
SPS	Solid Phase Spectrophotometry
SPs	Surface plasmons
SWCNTs	Single-walled carbon nanotubes
SWV	Square wave voltammetry
Ta	Tantalum
TCPP	Meso-tetra (4-carboxyphenyl) porphine
TGA	Thioglycolic acid
TiNTs	Titania nanotubes
TP^+^	2,4,6-triphenylpyrylium ion
TPY	2,4,6-triphenylpyrylium ion stabilized inside zeolite matrix
Trp-GR	Tryptophan-functionalized graphene nanocomposite
TS-PANI	Tetragonal star like Polyaniline
UA	Uric acid
UNCD	Ultrananocrystalline diamond
VNIR	Visible and near-infrared
ZnSa	Zinc–salophen
Zr[Fe(CN)_6_]	Zirconium hexacyanoferrate (II)
β-CD	β-cyclodextrin
